# Lactose intolerance at a day care center in the municipality of Duque de Caxias, Rio de Janeiro, Brasil

**DOI:** 10.1186/1939-4551-8-S1-A284

**Published:** 2015-04-08

**Authors:** Isaac Azevedo Tenorio, Aderbal Sabra, Selma Sabra

**Affiliations:** 1 Unigranrio, Brazil

## Objectives and study

Lactose intolerance has a variable prevalence in different parts of the world. The differences may be explained by the diverse eating habits of each population, allowing over the years a selection of individuals with and without the ability to digest lactose – ontogenetic variation. The objective of this study is to identify the prevalence of lactose intolerance and their presenting main symptoms, developed by children at a Day care center in the municipality of Duque de Caxias, Rio de Janeiro, Brazil, after an oral lactose tolerance test.

## Methods

Oral lactose tolerance test was done in all children at the daycare center. At the end of the test a questionnaire was held to identify symptoms. A hundred children were analyzed (61% male and 39% female), between 2 and 13 years (30% from 2 to 4 years; 37% from 5 to 7 years; 24% from 8 to 10 years; and 9% from 11 to 13 years).

## Results

The blood test showed 52% of the children with positive results for lactose intolerance and 48% were negative. From the questionaires answers were gotten in 64% of the patients. Lactose intolerance (positive test with symptoms) was present in 20.3% of the samples. Inconclusive results (positive test without symptoms and negative test with symptoms) reached 42.2% of the samples. Healthy patients represent 37.5% of the studied population. Among the symptoms, diarrhea was the most prevalent (50%), followed by abdominal pain and headache (20%), and flatus/abdominal distension (5%). Children between 5 and 7 years were the more symptomatic.

## Conclusion

Lactose intolerance was present and frequent among children at the evaluated Day care centerThe significant prevalence of this disease makes indispensable the referal to a Gastroenterology Unit all patients under the suspicious of lactose intolerance, to get the proper diagnosis and treatment as well as the proper diet orientation.

